# Medical and recreational cannabis use in patients undergoing one- or two-level lumbar spine fusion correlated with postoperative outcomes

**DOI:** 10.1016/j.xnsj.2025.100773

**Published:** 2025-07-18

**Authors:** Andrea T. Kwaczala, Matthew J. Solomito, Caitlin McCracken, Heeren Makanji

**Affiliations:** a Hartford HealthCare Bone and Joint Institute, 31 Seymour St, Hartford, CT, 06106, United States; b Orthopedic Associates of Hartford, Hartford, CT, United States

**Keywords:** Cannabis, Opioids, Complications, Spine fusion, Medical cannabis, Risk population

## Abstract

**Background:**

Cannabis use in the United States has become increasingly prevalent due to the legislation leading to decriminalization in several states; with increased social acceptance, patients are more willing to disclose cannabis use. Few studies have explored how cannabis may influence a patient’s recovery following elective lumbar fusion. Therefore, the purpose of this study was to investigate how cannabis use was associated with patient recovery following elective lumbar fusions.

**Methods:**

This retrospective single institution study included patients ages 35 through 80 years old who had undergone an elective single- or 2-level lumbar fusion between January 2021 and June 2024. Patients were placed into 1 of 3 study groups based on cannabis use, medical cannabis (MC), recreational cannabis (RC), and nonusers (NU). Differences in patient outcomes were assessed through univariate comparison and multivariate regression analyses.

**Results:**

627 patients were included, 129 (20.3%) admitted to cannabis use, 42 (32.5%) used medical cannabis and 87 (67.5%) used recreationally. Cannabis users were younger than NU (*p*<0.001) but reported increased pain (*p*=0.026) and required more opioids (*p*=0.017). Surgical site infections at 90 days (SSIs) were significantly greater in the MC group (*p*<0.001).

**Conclusions:**

Cannabis use and type of usage had an impact on patient-reported outcomes, pain level, and measures of surgical success. The MC group had significantly higher opioid consumption and SSI rates at 90 days compared to nonusers and recreational groups. Therefore, this study suggests cannabis use may influence postoperative recovery following elective spine fusion. Additionally, medical cannabis users may be a high-risk group not previously identified in the literature.

**Level of Evidence:**

III.

## Graphical abstract

**Figure:**
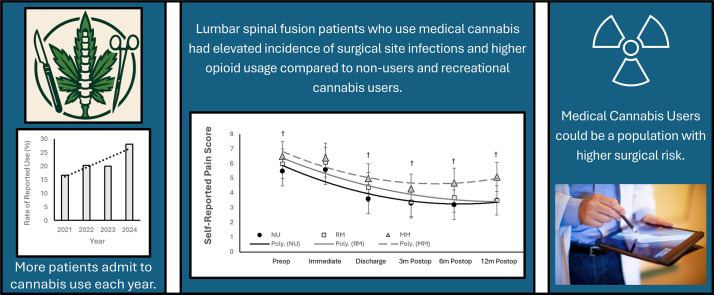

## Background

Cannabis is the most popular psychoactive substance in the world, with nearly 3.8% of the world’s population (183 million people) using it regularly, and its use in the United States has become increasingly prevalent due to the shifting public sentiment and legislation leading to decriminalization in several states [1]. Recent studies have shown that for Americans below the age of 35, cannabis use now outpaces tobacco use, and this trend is expected to continue given that the legal landscape has shifted greatly in favor of allowing cannabis usage for both medical and recreational uses [2]. With the increased tolerance and social acceptance, patients are now seemingly more willing to disclose their cannabis use. Therefore, there is a clear and present need to better understand how cannabis use may influence the postoperative recovery course of patients following surgical procedures.

Studies have shown therapeutic effects of cannabis use with chronic pain, sleep issues, and chemotherapy-induced nausea [3]. Use of cannabis in orthopedic surgeries has become increasingly more common [[4], [5], [6]]. Managing pain is especially relevant for orthopedic procedures, but studies show contradictory results where Medina et al. looked at a cohort of over 900 patients who underwent orthopedic procedures and observed that recreational marijuana users had less pain and better mobility compared to a matched cohort [7]. However, Lui et al. reported more perioperative pain, sleeplessness, postoperative pain and increased postoperative opioid consumption [8]. Additionally, studies have also shown negative effects associated with impaired cognition, and increased risks of cardiovascular and cerebrovascular events [[9], [10], [11]]. Therefore, competing and at times contradictory results currently in the literature make it difficult to draw meaningful conclusions to develop strategies towards risk mitigation in cannabis users undergoing elective orthopedic procedures.

One unique study provided in vitro data that suggested that cannabis may have a synergistic effect with opioids, that when used in conjunction, provided a similar analgesic effect at a lower opioid dose [12]. Although opioids are a mainstay means of controlling pain following orthopedic spine procedures, opioids are associated with a number of adverse effects and a risk of dependence, and as of 2019, there were over 50,000 opioid-related deaths in the United States [13]. Simultaneous to the dramatic increase in opioid use and opioid related mortality, there has been an expansion of the legalization of cannabis in the United States. There is evidence to suggest that cannabis use may reduce statewide opioid prescriptions [[14], [15], [16]]. Contradicting these findings, opioid consumption duration and amount increased after musculoskeletal injury among self-reported marijuana users [17].

Given the current information surrounding the use of cannabis and its influence on postoperative outcomes and a patient’s health, there remains a paucity of information describing how cannabis use prior to elective spine fusions may influence postoperative recovery. Therefore, the purpose of this study was to investigate the associations between of cannabis usage on clinical outcomes, patient reported outcomes, and pain management requirements after elective lumbar fusions. It was hypothesized that there would be no differences in outcomes between medical and recreational cannabis.

## Materials and methods

This was a retrospective single institution study conducted at a tertiary specialty orthopedic surgical hospital in Hartford, Connecticut. Recreational use of cannabis has been legal in the state of Connecticut since 2021, leading to increased tolerance and social acceptance by the patient population who are more willing to disclose their cannabis use. The study was approved by the organization’s Institutional Review Board. Patients between the ages of 35 and 80 years old and had undergone an elective single- or 2-level lumbar fusion between January 2021 and June 2024 were included in this study. Patients were excluded from this study if they had not completed their preoperative evaluation at our institution’s preoperative optimization clinic, and completed their preoperative patient reported outcomes (PROs). It is important to note, that part of the preoperative visit requires the completion of preoperative PROs unless there is a specific reason for the patient not to complete them, which results in nearly 98% compliance for preoperative PRO completion. Patients were excluded if the surgery was a result of trauma or pathological condition (eg, cancer). Prior surgery, other than spine surgery, was not an exclusion criterion. They were also excluded if they had a known opioid addiction, were being actively treated by a pain management specialist, or had a history of illicit drug use. Patients using only CBD products without a THC element were also excluded from this study. All patients indicating cannabis use during their preoperative visit were advised to discontinue use at least 2 weeks prior to surgery.

Patients were stratified into 1 of 3 study groups based on the patient’s self-disclosure of cannabis use: medical cannabis users (MC), recreational cannabis users (RC), and nonusers (NU). Patients were surveyed prior to surgery during preoperative clinics for cannabis use [18]. Patient and surgical data included patient age, sex, race, alcohol usage, tobacco usage, insurance payer type, surgical approach, levels fused, and year of surgery. Clinical measures included: in-hospital length of stay, postoperative ambulation distance on day of discharge, inpatient opioid use measured in morphine milligram equivalents (MME), surgical time, and recovery time in the Post anesthesia care unit (PACU). Patient-reported outcomes included pain, collected using the numerical pain scale [19], at the preoperative visit, immediately following surgery, at time of discharge, at 3-month, 6-month and 12-month follow up appointments. The Oswestry Disability Index (ODI) scores [20] were also collected preoperatively, 3, 6, and 12 months postoperatively, as well as patient satisfaction 6 months post fusion [21]. Complications including return to the Emergency Department (ED), unplanned readmittance (readmit), return to operating room (RTOR), deep surgical site infection (SSI) within 90 days of the index procedure were collected.

Group comparisons were performed across the medical, recreational cannabis use and nonuser control groups for overall differences in patient demographics, surgical types/technique, and postoperative outcomes using single factor ANOVAs, chi-square or Fisher exact tests. If overall group differences were significant, a post hoc pairwise test was performed using the Tukey honestly significant difference (HSD). To determine risk factors that contributed to surgical site infection risk, a multivariate regression analysis was performed to understand how potentially confounding factors (ie, age, gender, tobacco, alcohol use, surgical complexity, length of hospital stay) were associated with surgical site infection risk. In this case-control study where the actual risk cannot be directly calculated, the odds ratio (OR) of an event occurring in 1 group (eg, exposed to a risk factor) compared to the odds of the event occurring in another group (eg, not exposed) was determined. An OR greater than 1 suggests the risk factor is associated with increased odds of the outcome, while an OR less than 1 suggests a protective effect. Confounders of surgical site infection risk were chosen based on univariate analysis and known factors that influence surgical outcomes (eg, tobacco use). To determine whether cannabis raised the risk of infection, the nonuser group was used as the reference group and odds ratios were compared to look at the relative increase in incidence or infection rate where a p-value less than 0.05 was considered significant. All statistics were performed using STATA SE version 17 (StataCorp LLC, College Station, TX).

## Results

**Fig. 1:**
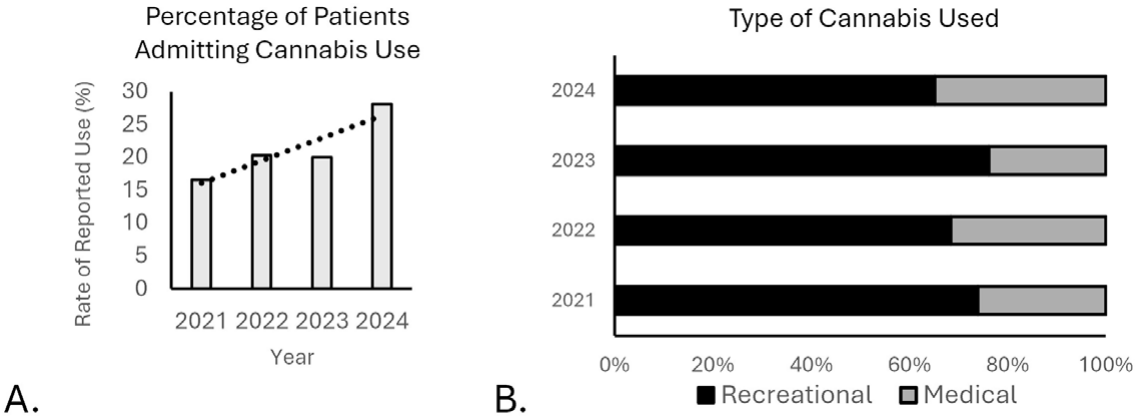
Patients reported cannabis usage in an orthopedic surgical population. (A) The percentage of patients admitting to cannabis use increased since 2021. (B) Types of cannabis use shifted towards medical since 2021.

**Fig. 2:**
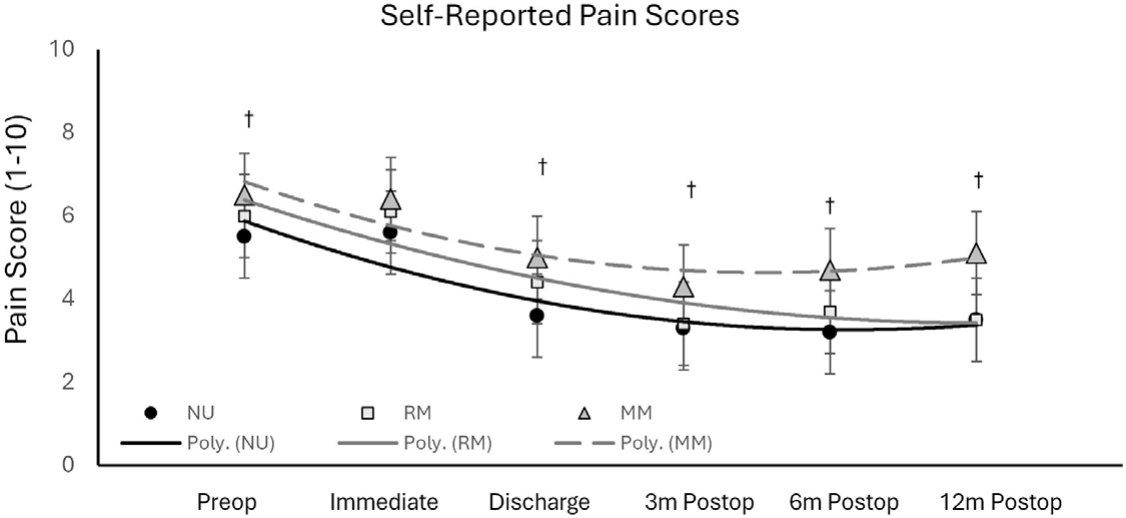
Self-Reported Pain before and after Recovery from Spinal Fusion. A) Pain scores were significantly higher in MC (medical cannabis) users at all time points except immediately after the surgery. Pain levels subsided in both NU (nonusers) and RC (recreational) users but rebounded towards the preoperative levels by 12 months in the MC group (nonusers, p<0.05, †).

Data indicated a substantial increase in cannabis usage year-over-year from 2021 to 2024, and a greater variation in type of cannabis used (Fig. 1). A total of 627 patients were included in this study, of which 129 (20.3%) admitted to cannabis use at the preoperative appointment; of those admitting use, 42 (32.5%) used medical cannabis and 87 (67.5%) used recreationally. There were no significant differences in demographics, surgical approach or levels fused among study groups with the noted exception that cannabis users were younger than nonusers (p<0.001, Table 1). It was also noticed that recreational users were predominately male compared to the other study groups (p=0.028). There were minimal differences for surgical time, PACU recovery time, and in-hospital length of stay (Table 2). Although not significant, there was a trend towards reduced ambulation distance on day of discharge among the study groups with the MC group walking the shortest distances (p=0.19, Table 2). This happened despite similar pain scores in all groups immediately after surgery (p>0.05, Fig. 2).

**Table 1:** Comparison of patient demographics across study groups.

	NonUsers	Recreational	Medical	P-value
N	498	87	42	
Age	65.1 ± 10.8	56.6 ± 14.2†	58.8 ± 13.5†	<.001
BMI	31.0 ± 6.3	30.9 ± 5.7	31.8 ± 7.4	0.732
Sex				
Male	234 (44.6%)	52 (59.7%) †‡	18 (42.9%)	0.028
Female	291 (55.4%)	35 (40.3%) †‡	24 (57.1%)	
Race				
African American	32 (6.1%)	2 (2.3%)	1 (2.4%)	0.380
Caucasian	437 (83.2%)	72 (82.8%)	37 (88.1%)	
Other	56 (10.7%)	13 (14.9%)	4 (9.5%)	
Insurance				
Commercial	13 (2.5%)	2 (2.3%)	1 (2.4%)	0.023
Medicaid	12 (2.3%) ‡	5 (5.8%) ‡	6 (14.3%)	
Medicare	458 (87.2%) ‡	73 (83.9%) ‡	31 (73.8%)	
Workers comp	40 (7.6%)	6 (6.9%)	4 (9.5%)	
Other government	2 (0.4%)	1 (1.2%)	0 (0.0%)	
Lifestyle				
Alcohol use current	306 (61.4%)	58 (66.7%)	25 (59.5%)	0.168
Alcohol use former	63 (12.7%)	18 (20.7%)	9 (21.4%)	
Tabacco use current	26 (5.2%)	14 (16.1%)	5 (11.9%)	0.364
Tabacco use former	202 (40.6%)	54 (62.2%)	22 (52.4%)	
Approach				
Anterior	81 (15.4%)	15 (17.2%)	4 (9.5%)	0.556
Combine	133 (25.3%)	14 (16.1%)	7 (16.7%)	
Oblique	52 (9.9%)	12 (13.8%)	8 (19.1%)	
Posterior/Trans.	259 (49.3%)	46 (52.9%)	23 (54.7%)	
Level				
1	395 (75.2%)	60 (68.9%)	35 (83.3%)	0.197
2	130 (24.7%)	27 (31.1%)	7 (16.7%)	

BMI, body mass index; NU, nonuser group, RC, recreational cannabis group, MC, medical cannabis group.

† significantly different than the NU group.

‡ significantly different than the MC group.

**Table 2:** Comparison of postoperative clinical outcomes across study groups.

Inpatient metrics (mean ± standard deviation)				
	Nonusers	Recreational	Medical	P-value
Surgical time (min)	175 ± 63	196 ± 79 †	186 ± 88	0.031
PACU recovery time (min)	142 ± 84	154 ± 166	130 ± 44	0.414
Length of stay (days)	3.1 ± 1.2	3.4 ± 2.3	3.7 ± 1.6†	0.034
Opioid use (MME)	135 ± 132‡	182 ± 157†^,^‡	264 ± 288†	0.017
Ambulation distance (ft)	198 ± 228	162 ± 185	135 ± 150	0.195

Complication Rates (% of group)				
	Nonusers	Recreational	Medical	P-value
ED utilization (90 days)	8.0%	8.0%	4.2%	0.747
Readmit (90 days)	4.4%	4.6%	4.8%	0.993
SSI (90 days)	1.0% ‡	1.1% ‡	9.5% †	<.001

NU, nonuser group; RC, recreational cannabis group; MC, medical cannabis group; MME, morphine milligram equivalent; ODI, oswestry disutility index; MCID, minimal clinically important difference; ED, emergency department; SSI, surgical site infection.

† significantly different than the NU group.

‡ significantly different than the MC group.

There were significant differences in pain among study groups from the initial preoperative assessment through 1 year after procedure (Fig. 2). Recreational and medical users reported greater levels of pain preoperatively and on day of discharge compared to nonusers (p*=0*.026 and p*=0*.001, respectively). Medical cannabis users reported significantly higher pain levels at 3, 6, and 12 months postoperatively compared to nonusers (p*=0*.040, p*=0*.004, and p*=0*.007, respectively). There was no significant difference in pain levels between nonusers and recreational users following discharge, or at postoperative time points 3, 6, or 12 months. Data indicated that there was a significant difference in the amount of in-hospital opioid consumption among study groups (p*=0*.017). Medical cannabis users consumed 96% more opioids than nonusers (p<0.001), and 45% more than recreational users (p*=0*.017). Recreational users were noted to consume 29% more opioids than nonusers (p*=0*.029).

Regarding postoperative complications, the results indicated that there were no differences in ED utilization or readmission rates; however, there was a significant difference in surgical site infections in the MC group compared to the other study groups (Table 2). At large, cannabis users had a 3.9% surgical site infection (SSI within 90 days) compared to nonusers that had a 0.9% infection rate. Medical cannabis users had a significantly greater SSI within 90 days than any group at 9.5% (p<0.001) and the recreational users surgical site infection rate was in line with the nonuser group at 1.1% (p>0.05). A multivariate logistic regression analysis controlling for possible confounding factors was used to further understand the relationship between the high surgical site infection rate noted in the MC group (Table 3). This analysis controlled for age, sex, tobacco use, alcohol use, surgical levels, and length of stay in the hospital. Medical cannabis was the only independent factor that influenced the incidence of surgical site infections other than age. Results of the regression analysis indicated that medical cannabis use increased the risk of surgical site infections by 8-fold (p*=0*.007). It is important to note that the regression analysis only explained 18.4% of the SSI risk.

**Table 3:** Comparison of infection rate at 90-day postindex procedure among study groups.

	Study group	Incidence	P-value	Odds ratio	95% confidence interval
SSI (within 90 days)	Nonusers	5 (1.0%)	–	Ref.	Ref.
	Recreational	1 (1.1%)	0.644	0.58	0.1–5.9
	Medical	4 (9.5%)	0.007	8.1	1.8–36.7
SSI (90 days) controlling for age			0.015	0.93	0.9–1.0
SSI (90 days) controlling for gender			0.219	0.40	0.1–1.7
SSI (90 days) controlling for tobacco use			0.935	1.04	0.4–2.8
SSI (90 days) controlling for alcohol use			0.384	1.48	0.6–3.9
SSI (90 days) controlling for surgical level			0.081	3.68	0.9–15.9
SSI (90 days) controlling for length of stay			0.664	1.09	0.7–1.6

Ref., reference group for comparison against other study groups, nonusers, Pseudo R^2^ value = 0.184.

At the preoperative appointment, the ODI score was 17% higher in the medical cannabis users compared to recreational users suggesting these patients believed they were worse off than the other 2 groups. There were significant differences in PRO scores at both baseline and 3 months postfusion. At the 3-month post operative follow-up, all patients had improved self-reported outcomes (lower ODI scores) however the MC users still were 48% higher compared to nonusers and 25% higher than RC users (p<0.001). There was no difference in patient-reported satisfaction scores, where nonusers reported 83% satisfaction, recreational users were 81% and medical users were 74% satisfied with their recovery (p*=0*.395). All groups reached an MCID between 55 and 60% MCID scores (NU:56%, RC: 60%, MC:55%, p*=0*.832).

## Discussion

This study demonstrated important differences in surgical outcomes after elective spinal fusion among patients who reported using cannabis. The type of cannabis use differed in the patient-reported outcomes, pain level, and measures of surgical success. In general, medical cannabis users had suboptimal recovery when compared to the recovery of nonusers, but there were limited differences in recovery between recreational users and nonusers. Overall, medical cannabis users reported higher pain, required more opioids, and ambulated less than nonusers and had lower self-reported improvements after surgery than the other 2 groups, even though medical cannabis users were 8 years younger on average. It was also noted that medical cannabis users believed their functional status, as measured by the ODI, was worse than any of the other groups.

The most striking finding of this study was that the medical cannabis users had a significantly higher incidence of surgical site infections compared to nonusers and recreational users which indicates that medical cannabis might be a significant risk factor for poor outcomes after elective single- or 2-level lumbar fusions. Logistic regression revealed that patients using recreational cannabis were at no greater odds of developing a postoperative medical complication compared to the nonuser group, but medical cannabis users had an increased rate of infection. Contradictory to outcomes in the literature, where older patients tend to have higher risk of complications, this patient population showed that the younger patients were at greater risk of SSI within 90 days. It was also confirmed that length of stay did not independently contribute to increased infection rates. However, it is also important to temper this finding in that the numbers of patients with a surgical site infection were low overall within a relatively small subsample. Additionally, the confidence interval around the odds ratio was quite wide, further demonstrating the variability within this finding. Therefore, while significant, additional directed research is necessary to better understand the association between medical cannabis use and postsurgical infection risk. Additional research into this potential at-risk population is warranted, possibly looking at medical cannabis use as a marker of suboptimal healing potential.

Longitudinal characterization of patient group’s reported pain levels and opioid consumption helped to identify a clear difference in the MC recovery. Both NU and RC users had lowered reported pain levels at the 1 year postoperative follow up. Unfortunately, the MC group rebounded and had reported pain levels similar to the level of pain they were experiencing prior to surgery. It has been shown that preoperative pain levels are a risk factor for opioid dependence in orthopedic surgical care [22]. There is evidence to suggest that medical cannabis use can lead to worse pain when used chronically [23] which was evident in their higher doses of MMEs during the in-hospital recovery period. It is possible that the medical cannabis group were suffering from a higher level of chronic pain before the surgery as both their pain levels and reported ODI function before surgery was worse than both recreational users and nonusers.

As this study demonstrated, MC patients required more MMEs during the immediate postoperative period and had higher reported pain levels. Unsurprisingly, the increased pain levels were significantly correlated to opioid consumption which was 18 times greater in the MC group compared to nonusers which is consistent with previous reports [24,25]. Also concerning is that opioids prescribed during and after orthopedic surgery may trigger long-term use in patients and could be problematic, especially in patients who are opioid tolerant [26]. Consequences of overuse include increased mortality and morbidity following orthopedic surgery [24,25,27]. These results suggest that continued and/or extensive use of cannabis products taken by those in the MC group may increase tolerance to the analgesic effects of the narcotic medication [28,29].

Alcohol and/or tobacco use was not a predictor of increased MME usage, which is somewhat contrary to previously published studies suggesting a synergistic effect between tobacco and cannabis use [30]. It has been reported that spinal fusion patients who had elevated preoperative opioid levels were more likely to continue to use opioids after 1 year of use [31]. However, additional directed research is necessary to better understand how dosing and route of administration of medical cannabis may influence opioid consumption and pain reporting.

It was clear that medical cannabis did not appear to work for long term pain management in this patient population compared to those without a prescription. Self-reported pain scores could be used to isolate cases of medication misuse including any they may be self-prescribing to manage pain. This work is contradictory to others who found synergistic effects of cannabis with opioids, showing cannabis users required less opioid pain management [16]. It is possible increased cannabis use, or other pain management medications could be linked to hyperalgesia, increased sensitivity to pain [32,33]. Clinicians should consider how pain is assessed, find alternatives for pain management, and help those patients who are in chronic pain [34,35].

This study is not without limitations. This study has a relatively small sample of cannabis users. Additionally, this study was retrospective and thus by design is limited, especially since cannabis use was based on patient self-disclosure, and no information about the how often the patient used, dosing, or route of intake was able to be ascertained from the data. Additionally, all patients indicating cannabis use during their preoperative visit were advised to discontinue use at least 2 weeks prior to surgery; however, this was also self-disclosed and there was no way to be sure patients discontinued use. There are concerns of patient withdrawal during this preoperative window which could negatively impact patient recovery; the effects of withdrawal on orthopedic procedures requires additional research. Finally, the study population was relatively homogenous in its demographic and socio-economic description, and thus the results of this work may not be fully generalizable to other studies with greater variation within its population.

In conclusion, the use of cannabis was found to influence outcomes following elective, 1- or 2-level lumbar fusions. The results of this study suggest that cannabis use, especially medical cannabis use, may be associated with increased pain reporting, increased opioid consumption, and potential for increased risk of surgical site infection after elective lumbar fusion.

## Funding

This study did not receive any external funding.

## Declaration of competing interest

The authors declare that they have no known competing financial interests or personal relationships that could have appeared to influence the work reported in this paper.
